# Intentional Foreign Body Ingestion: A Complex Case of Pica

**DOI:** 10.1155/2019/7026815

**Published:** 2019-02-06

**Authors:** D. Guinan, T. Drvar, D. Brubaker, M. Ang-Rabanes, J. Kupec, P. Marshalek

**Affiliations:** ^1^West Virginia University Department of Behavioral Medicine and Psychiatry, General Psychiatry Resident PGY-2, USA; ^2^West Virginia University, USA; ^3^West Virginia University School of Medicine, MS-4, USA; ^4^West Virginia University Department of Behavioral Medicine and Psychiatry, Assistant Professor, USA; ^5^West Virginia University Department of Medicine Section of Digestive Diseases, Assistant Professor, USA; ^6^West Virginia University Department of Behavioral Medicine and Psychiatry, Medical Director, USA

## Abstract

Intentional ingestion of foreign objects, a form of self-injurious behavior, is rarely discussed in the medical literature but often requires extensive evaluation, management, and resources. It can be especially problematic for gastroenterologists, who are often consulted for removal of the foreign body. Pica is the psychiatric diagnosis for intentional ingestion of nonnutritive objects and is most commonly seen in prison inmates and those diagnosed with intellectual disability or psychiatric illness. This case report presents a challenging case of pica, highlighting the complexity involved in diagnosis and the need for early psychiatric intervention. It also aims to provide a general review of the literature and practical recommendations to assist with managing this form of self-injurious behavior in the inpatient setting. Collaborative efforts among specialties in addition to primary prevention are vital to successful management of these patients.

## 1. Introduction

Intentional foreign body ingestion is often challenging and frustrating to treat, demanding a considerable amount of time and resources. In many cases, foreign body ingestion occurs repeatedly in a small population of patients and can consume a large percentage of healthcare-related costs. This was demonstrated in a retrospective case study [1] in which 305 cases of intentional foreign body ingestion were associated with only 33 patients. Psychiatric diagnoses were identified in 79 percent of these patients and total cost for management of the cases totaled over $2 million. Elucidating factors contributing to these behaviors could be beneficial to specialists removing the objects to help prevent recurrence and facilitate more cost-efficient and effective care.

Intentional ingestion of foreign bodies has relatively little representation in the medical literature but is most commonly reported by specialists in emergency medicine, general surgery, and gastroenterology [1–4]. These groups tend to focus on the acute management and technique of foreign body removal, particularly the endoscopic techniques that revolutionized treatment approach. In contrast, reports exploring the psychiatric evaluation of these ingestion behaviors are scant. We present a case of repetitive intentional foreign body ingestion which demonstrates psychiatric complexity as well as diagnostic and treatment challenges associated with this self-injurious behavior.

## 2. Case Report

**Figure 1:**
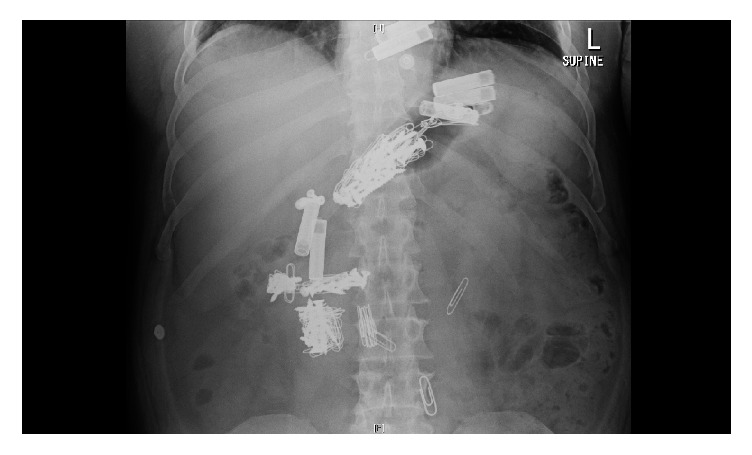
Abdominal X-ray showing multiple ingested substances later identified as 50 paperclips, 50 screws, eight batteries, and seven razor blades covered in paper.

A 39-year-old male presented to the emergency department after intentional ingestion of multiple objects including 50 paperclips, 50 screws, eight batteries, and seven razor blades covered in paper (Figure 1.). His past psychiatric history included major depressive disorder, generalized anxiety disorder, posttraumatic stress disorder, borderline personality disorder, and pica with a history of more than twenty admissions for ingestion behaviors often requiring endoscopic retrieval. Abdominal radiograph confirmed the presence of multiple metallic objects in the stomach and small intestine which were removed endoscopically by the gastroenterologist. The patient was then admitted to the hospital's adult inpatient psychiatric unit for further care.

The patient cited anxiety and an empty prescription for alprazolam as the primary trigger leading to the ingestions. Multiple psychotropic medications, including antidepressants (sertraline, citalopram, escitalopram, mirtazapine, and bupropion), mood stabilizers (lithium and Depakote), antipsychotics (olanzapine, haloperidol, and aripiprazole), and benzodiazepines (clonazepam and lorazepam), failed to control his ingestion behaviors. His explanations for the ingestions have varied (e.g., coping mechanism for prior sexual trauma, relieving impulses, and getting out of “difficult situations”, i.e., jail) but are always nonsuicidal in nature. He denies symptoms of depression but reports multiple symptoms of anxiety including restlessness, feeling tense, and having difficulty with sleep. He denies auditory or visual hallucinations and had no outward signs of paranoid thinking.

During admission, the patient was restarted on alprazolam 2 mg three times daily in conjunction with intensive therapy including Dialectical Behavior Therapy with components of mindfulness, distress tolerance, emotion regulation, and stress management skills. While on the unit, he swallowed a clock battery, screws from the toilet, and a colored pencil. He blamed the ingestion on anxiety and concern that unit staffs were angry with him. He denied the ingestions were a suicide attempt. The patient was subsequently sent back to the emergency department for endoscopy and the objects were successfully removed. He ultimately left against medical advice and no medication changes were made.

The patient was admitted for intentional ingestion four more times that month. During his most recent hospitalization, there was concern for misuse of his alprazolam prescription and he was weaned off and started on venlafaxine 37.5 mg and hydroxyzine 25 mg three times daily as needed for anxiety. He was discharged into a partial hospitalization program for daily psychotherapy groups and medication management. At the time of this writing, he has not been readmitted for pica.

## 3. Discussion

The Diagnostic Statistical Manual of Mental Disorders 5th Edition defines pica as the persistent eating of nonnutritive, nonfood substances over a period of one month which is inappropriate to the developmental level of the individual and not part of a socially or culturally normative practice. The symptoms must also be severe enough to warrant clinical attention [5]. It is often associated with micronutrient deficiencies (especially iron) and is frequently seen during pregnancy or in those with intellectual disability. Commonly ingested substances include ice, dirt, clay, starch, paper, hair, chalk, and metal artifacts which can occasionally require surgical removal [6]. Treatment of pica involves replacing micronutrients in those with deficiencies or implementing behavioral interventions (e.g., aversive stimuli, overcorrection, and reinforcement) in those with developmental disabilities [7]. In those with comorbid psychiatric illness or in populations where the behavior is used for secondary gain (e.g., prison populations), treatment becomes more challenging due to diagnostic complexity.

Four main diagnostic subgroups of psychiatric diagnoses are associated with intentional foreign body ingestion including malingering, psychosis, pica, and personality disorder [8]. Other psychiatric illnesses including obsessive compulsive disorder and autism spectrum disorder have been implicated in the behavior [9, 10]. Nationwide epidemiologic studies investigating the incidence of intentional foreign body ingestion have yet to be performed; however, one retrospective case series examining ingestions in adults of a lower socioeconomic population found that 92% of the ingestions were intentional and 85% involved patients with psychiatric comorbidities [11]. As highlighted by our case report, a patient may demonstrate several psychiatric comorbidities that accompany their pica.

Treating pica often requires a multidisciplinary approach. Once micronutrient deficiencies have been ruled out, screening for and treating the underlying psychiatric diagnosis may help minimize the intentional ingestion behavior. For the gastroenterologist faced with a patient who intentionally ingests, early involvement of the psychiatric service can facilitate diagnostic clarification. Neuropsychologic testing may assist with identifying mild intellectual disability associated with pica which responds best to behavior modification therapy. Trials of typical and atypical antipsychotics in patients with pica related to an underlying psychotic disorder have been successful in some reports but ineffective in others [12, 13]. Pica related to obsessive compulsive disorder has shown good response to selective serotonin reuptake inhibitor (SSRI) therapy in several cases [14, 15]. In terms of personality disorders, pica is most frequently seen in borderline personality disorder and is proposed to be a form of affect regulation, much like cutting behaviors. Psychiatric admission solely for intentional ingestion related to a personality disorder has not been shown to prevent recurrence of the behavior and may actually lead to escalation of swallowing [16].

Endoscopic retrieval of foreign bodies is costly and, though generally considered a low-risk procedure, can be associated with major complications including bleeding and perforation. Considering repeated ingestions are often seen in patients with comorbid psychiatric illness, prevention of ingestions should be the therapeutic goal of all specialists involved in the care of these individuals. Specific prevention strategies that can be utilized during inpatient admission include the following:

- placing patients under constant visual observation status while admitted, whether this be through video monitoring or by having a sitter present at bedside;
- immediately removing potentially ingestible objects from patient's room;
- administering psychotropic medications when indicated;
- utilizing an individualized care plan to prevent reinforcement of behavior and mitigate readmissions and frequent imaging. For example, a specific care plan was implemented by both emergency department and inpatient medical providers in order to improve patient compliance with treatment and promote better outcomes of care. The care plan recommended early involvement of both gastroenterology and psychiatry services and avoidance of intravenous opioids and sedative/hypnotics due to concerns for medication misuse and addiction.

Additionally, a significant amount of frustration and countertransference can arise in healthcare providers when treating patients who repetitively ingest. It is important to remember patients who inflict self-harm, particularly through repetitive foreign body ingestion, have a right to treatment. Continued efforts must be made to best serve patients with pica and comorbid psychiatric diagnoses.

## 4. Conclusion

Pica is a diagnosis that can be associated with intentional foreign object ingestion and may overlap with multiple other psychiatric conditions. When addressing this behavior, it is important to promote a safe environment while simultaneously screening for and managing any concurrent mental illness. Elucidating and treating specific psychiatric comorbidity can be challenging, and collaborative care from multiple specialists is often required. Early involvement of both gastroenterologists and psychiatrists can prove helpful when determining the best and most comprehensive approach to treatment. Future work is needed to continue to identify successful strategies and specific care plans to meet the needs of these challenging patients.
